# Covid-19: predictive mathematical formulae for the number of deaths during lockdown and possible scenarios for the post-lockdown period

**DOI:** 10.1098/rspa.2020.0745

**Published:** 2021-05

**Authors:** Athanassios S. Fokas, Nikolaos Dikaios, George A. Kastis

**Affiliations:** ^1^ Department of Applied Mathematics and Theoretical Physics, University of Cambridge, Cambridge, UK; ^2^ Mathematics Research Center, Academy of Athens, Athens, Greece; ^3^ Department of Biomedical Engineering, University of Southern California, Los Angeles, CA, USA; ^4^ Centre for Vision, Speech and Signal Processing, University of Surrey, Guildford, UK

**Keywords:** mathematical modelling of epidemics, Covid-19, Riccati equation, integrable systems, inverse problems

## Abstract

In a recent article, we introduced two novel mathematical expressions and a deep learning algorithm for characterizing the dynamics of the number of reported infected cases with SARS-CoV-2. Here, we show that such formulae can also be used for determining the time evolution of the associated number of deaths: for the epidemics in Spain, Germany, Italy and the UK, the parameters defining these formulae were computed using data up to 1 May 2020, a period of lockdown for these countries; then, the predictions of the formulae were compared with the data for the following 122 days, namely until 1 September. These comparisons, in addition to demonstrating the remarkable predictive capacity of our simple formulae, also show that for a rather long time the easing of the lockdown measures did not affect the number of deaths. The importance of these results regarding predictions of the number of Covid-19 deaths during the post-lockdown period is discussed.

## 1. Introduction

Severe acute respiratory syndrome coronavirus 2 (SARS-CoV-2) is the third coronavirus to appear in the human population in the past two decades; the first was the severe acute respiratory syndrome coronavirus (SARS-CoV) that caused the 2002 outbreak; the second was the Middle East syndrome coronavirus (MERS-CoV) responsible for the 2012 outbreak. SARS-CoV-2 has now caused a pandemic, which poses the most serious global public health threat since the devastating 1918 H1N1 influenza pandemic that killed approximately 50 million people (in proportion to today's population, this corresponds to 200 million people). The unprecedented mobilization of the scientific community has already led to remarkable progress towards combating this threat, such as understanding significant features of the virus at the molecular level; see for example [1,2]. In addition, international efforts have intensified towards the development of specific pharmacological interventions, including clinical trials using old or relatively new medications and the employment of specific monoclonal antibodies, as well as novel approaches for the production of an effective vaccine. In particular, the US Food and Drug Administration has granted a conditional approval to the anti-viral medication remdesivir.^1^ Unfortunately, the combination of the anti-viral medications lopinavir and ritonavir that are effective against the human immunodeficiency virus has not shown any benefits [3]; similarly, for the combination of the anti-malarial medication hydroxychloroquine and the antibiotic azithromycin [4]. There are ongoing clinical trials testing the synthetic protein tocilizumab that binds interleukin-6 (this medication is often used in rheumatoid arthritis), as well as clinical trials involving the infusion of infected patients with plasma from individuals who have recovered from coronavirus disease (Covid-19) [5].

The scientific community is also playing an important role in advising policy-makers about possible non-pharmacological approaches to limit the catastrophic impact of the pandemic. For example, following the rigorous analysis in [6] of two possible strategies, called mitigation and suppression, for combating the epidemic, the UK switched from mitigation to suppression. Within this context, in order to design a long-term strategy, it is necessary to be able to predict important features of the Covid-19 epidemics, such as the final accumulative number of deaths. Clearly, this requires the development of predictive mathematical models.

In a recent paper [7], we presented a model for the dynamics of the accumulative number of individuals in a given country that are reported at time *t* to be infected by SARS-CoV-2. This model is based on a particular ordinary differential equation of the Riccati type, which is specified by a *constant parameter* denoting the final total number of infected individuals and by a *time-dependent function.* Remarkably, although the above Riccati equation is a nonlinear equation that is conditional on time-dependent coefficients, it can be solved in closed form. Its solution depends on the above parameter and function, as well as on a parameter related to the constant arising in integrating this equation. In the particular case that the associated time-dependent function is a constant, the explicit solution of the above Riccati equation becomes the classical *logistic formula*. It was shown in [7] that, although this formula provides an adequate fit for the given data, it does *not* yield sufficiently accurate predictions. In order to provide more accurate predictions, two novel formulae were introduced in [7], called *rational* and *birational*. The predictive capabilities of these formulae were established, first by comparing their predictions with real data and second by showing that a complicated deep learning algorithm did not perform better than these explicit formulae.

Here, we will show that similar expressions can also be used for determining the time evolution of the number, *N*(*t*), of deaths in a given country caused by Covid-19. The Riccati equation formulated in [7] is now specified by the parameter N_f_, denoting the final total number of deaths, and by the function *α*(*t*); this function and N_f_ model the effect of the basic characteristics of Covid-19 as well as the cumulative effect of the variety of different measures taken by the given country for the prevention and the treatment of this viral infection. In the case that *α*(*t*) is a constant, denoted by k, the solution of the above Riccati equation becomes the well-known logistic formula,

1.1 $$N(t)=\frac{N_{f}}{1+\beta {\text{e}}^{-kt}},$$

where β is the constant of integration. In the case that *α*(*t*) is the rational function kd/(1 + d*t*), the Riccati equation yields the rational formula; this expression is obtained from the logistic formula by simply replacing the exponential function with the function (1 + d*t*)*^−k^*, where d and k are constants. The birational formula is similar to the rational formula, but the associated parameters are different before and after a fixed time X; this constant parameter is either T or in the neighbourhood of T, where *T denotes the time that the rate of deaths reaches a maximum*. The point on the curve depicting *N* as a function of *t* corresponding to *t* = T is known as the *inflection point*.

It turns out that, in general, the birational formula yields better predictions than the rational, which in turn provides better predictions than the logistic. However, the birational formula can be employed only after the curve approaches its sigmoidal part (a precise criterion of when the birational can be used is discussed in §4). For this reason, since we only used data until 1 May 2020, at which time the epidemic in Germany had not yet reached the sigmoidal part of the curve, the birational formula could not be used for the epidemic in Germany.

**Figure 1.**
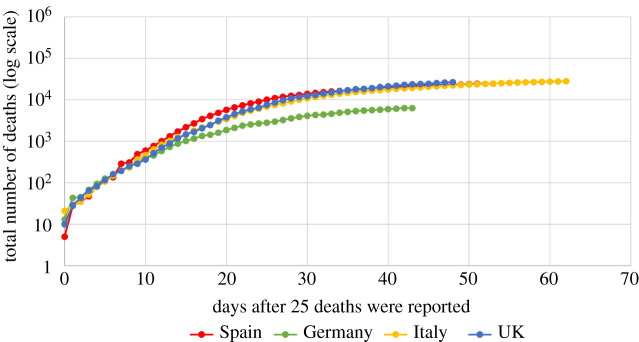
The total cumulative number of deaths in Spain, Germany, Italy and the UK, due to Covid-19, reported up to 1 May 2020, as a function of days after the day that 25 deaths were reported. (Online version in colour.)

Figure 1 depicts the total number of deaths as a function of time after the day that 25 deaths had occurred, for the epidemics in Spain, Germany, Italy and the UK. The reason for analysing the epidemics after the time that 25 deaths are reached is that the data are quite noisy before this stage (this is analogous to the fact that usually models for the number of individuals reported infected with SARS-CoV-2 consider data after the number of reported infected reaches 500).

In this work, we implemented the following tasks. (i) We determined the parameters specifying the logistic, rational and birational formulae (for Germany only the first two formulae were used) using the above data, namely until 1 May 2020. In this way, it became evident that each of these models *can* fit the data. (ii) We compared the predictions of these models for the following 122 days, namely until 1 September 2020. These comparisons show that the predictions for Spain, Italy and Germany via our novel formulae are truly outstanding, and for the UK quite good. (iii) We computed the time of the plateau as well as the value of *N* at the plateau using the logistic and our two formulae (for Germany only the logistic and the rational formulae were used), where *the plateau is defined as the point when the rate of deaths is 5% of the maximum rate*.

By implementing the above tasks, we found the following estimates for the dates that the plateau is reached as well as for the number of deaths when this occurs: Spain plateaus on 25 May 2020 with 27 090 deaths; Germany plateaus on 16 June 2020 with 8702 deaths; Italy plateaus on 19 June 2020 with 34 399 deaths; and the UK plateaus on 25 June 2020 with 38 487 deaths. As will be discussed below, these estimates are very accurate for the characteristics of the actual plateaux in the above four countries.

## 2. Results

Table 1 presents the model parameters and the plateau characteristics for the three different formulae, for Spain, Germany, Italy and the UK (for the epidemic in Germany, only the parameters and characteristics of the logistic and rational formulae are presented). The confidence intervals for each fitting parameter are also presented. The calculation of the inflection point requires the time that the derivative of *N* becomes maximum. To compute this point, we used the logistic formula (the other formulae yield similar results). For Spain, Germany, Italy and the UK the inflection point occurred at *t* = 28, *t* = 27, *t* = 35 and *t* = 32, respectively. This corresponds to 6 April, 15 April, 4 April and 15 April 2020, respectively.

**Table 1.** Model parameters and plateau characteristics for the logistic, rational and birational formulae for the epidemics in Italy, Spain and the UK. For the epidemic in Germany, only the parameters and characteristics of the logistic and rational formulae are presented. The confidence intervals for each fitting parameter are also presented.

	Italy	Spain	UK	Germany
logistic model				
N_f_	27 687	24 016	27 708	6906
	(26 979, 28 395)	(23 410, 24 622)	(27 129, 28 287)	(6680, 7124)
k	0.1188	0.1481	0.1533	0.143
	(0.1020, 0.1258)	(0.1384, 0.1578)	(0.1488, 0.1578)	(0.1357, 0.1503)
β	63.4044	65.7474	134.8143	51.2444
	(50.0735, 76.7353)	(49.2186, 82.2762)	(129.2112, 140.4174)	(43.7599, 58.7289)
T	35	28	32	27
plateau (days)	73	59	62	59
plateau (deaths)	27 390	23 765	27 433	6830
*R*^2^	0.9955	0.9955	0.9975	0.9979
RMSE	678	607	475	102
rational model				
N_f_	33 153	27 524	34 920	9021
	(31 764, 34 542)	(26 532, 28 516)	(34 267, 35 573)	(8197, 9845)
k	3.6299	3.8035	4.0954	3.7379
	(3.1668, 4.0930)	(3.2815, 4.3255)	(3.8357, 4.3551)	(2.9106, 4.5652)
β	4892.02	6372.60	23 496.07	451.2287
	(4317.43, 5466.60)	(5824.45, 6920.80)	(22 976.40, 24 0160)	(315.4700, 586.9874)
d	0.2455	0.2996	0.2997	0.12828
	(0.2120, 0.2790)	(0.2630, 0.3362)	(0.2546, 0.3448)	(0.1073, 0.1493)
plateau (days)	101	77	88	89
plateau (deaths)	31 976	26 581	33 886	8702
*R*^2^	0.9994	0.9994	0.9996	0.9994
RMSE	262	233	199	55
birational model				
k	6.7118	5.0583	4.4465	
	(5.4890, 7.9346)	(3.5627, 6.5539)	(3.265, 5.628)	
b	953.44	6532.56	39 666	
	(617.10, 1289.80)	(4187.6, 8877.5)	(36 475, 42 857)	
c	23 310	21 434	29 724	
	(20 820, 25 800)	(19 329, 23 539)	(28 914, 30 534)	
d	0.0572	0.1814	0.3000	
	(0.0085, 0.1059)	(0.0837, 0.2791)	(0.2662, 0.3338)	
k_1_	4.0093	4.6349	3.5263	
	(3.6935, 4.3251)	(4.1008, 5.1690)	(1.7963, 5.2563)	
b_1_	478.1700	97 887.64	27 864.22	
	(385.2837, 571.0563)	(7034, 12 541)	(20 965.32, 34 763.12)	
c_1_	31 737	21 766	38 492	
	(30 303, 33 171)	(20 367, 23 165)	(31 449, 45 535)	
d_1_	0.0893	0.1796	0.4354	
	(0.0779, 0.1007)	(0.1338, 0.2254)	(0.1081, 0.7627)	
plateau (days)	111	77	103	
plateau (deaths)	34 399	27 090	38 487	
*R*^2^	1.0000	0.9999	0.9998	
RMSE	60	82	148	

**Figure 2.**
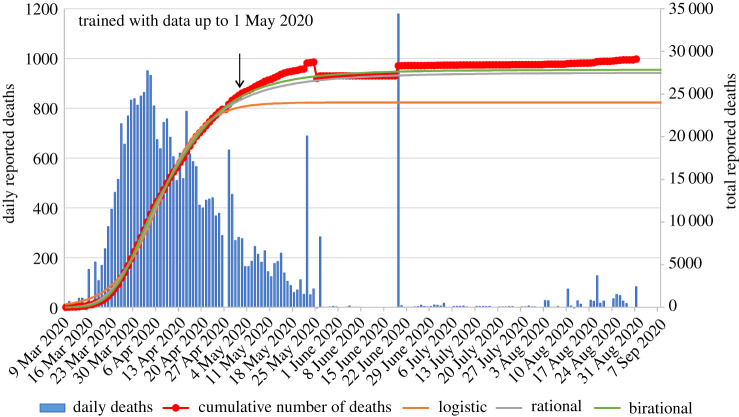
Comparison of the predictions of our formulae for the number of reported deaths for Spain using data only until 1 May 2020, versus the actual data until 1 September 2020 (predictions for 122 days). The data points with circle markers depict the cumulative number of deaths. The arrow indicates the last data point used for calibrating the formulae. Negative daily reported deaths are not displayed for clarity. The inflection point for Spain occurred on 6 April 2020. (Online version in colour.)

**Figure 3.**
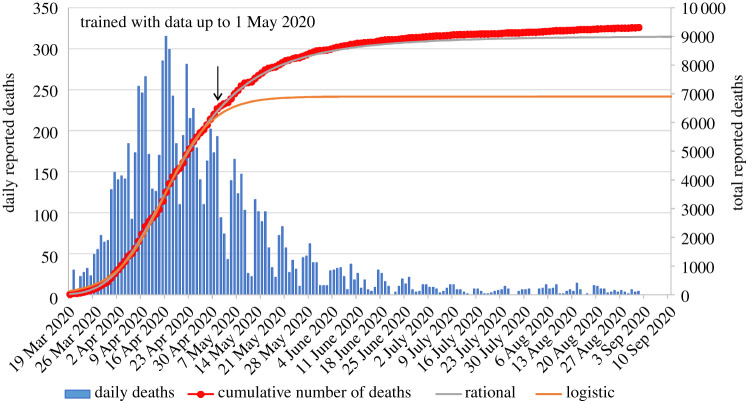
Comparison of the predictions of our formulae for the number of reported deaths for Germany using data only until 1 May 2020, versus the actual data until 1 September 2020 (predictions for 122 days). The data points with circle markers depict the cumulative number of deaths. The arrow indicates the last data point used for calibrating the formulae. Negative daily reported deaths are not displayed for clarity. The inflection point for Germany occurred on 15 April 2020. (Online version in colour.)

**Figure 4.**
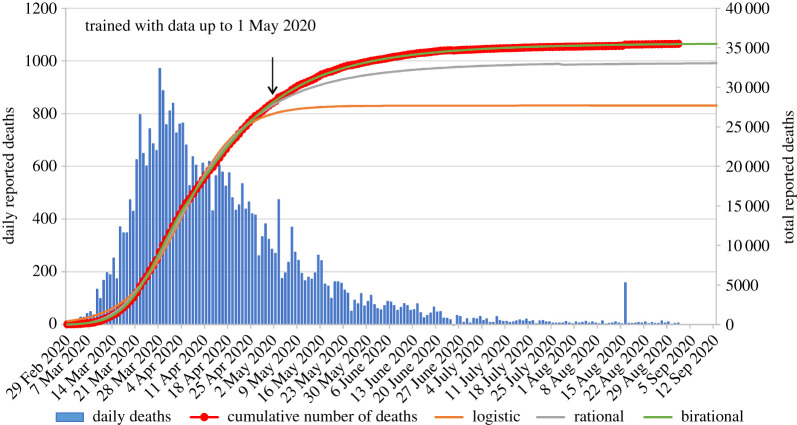
Comparison of the predictions of our formulae for the number of reported deaths for Italy using data only until 1 May 2020, versus the actual data until 1 September 2020 (predictions for 122 days). The data points with circle markers depict the cumulative number of deaths. The arrow indicates the last data point used for calibrating the formulae. Negative daily reported deaths are not displayed for clarity. The inflection point for Italy occurred on 4 April 2020. (Online version in colour.)

**Figure 5.**
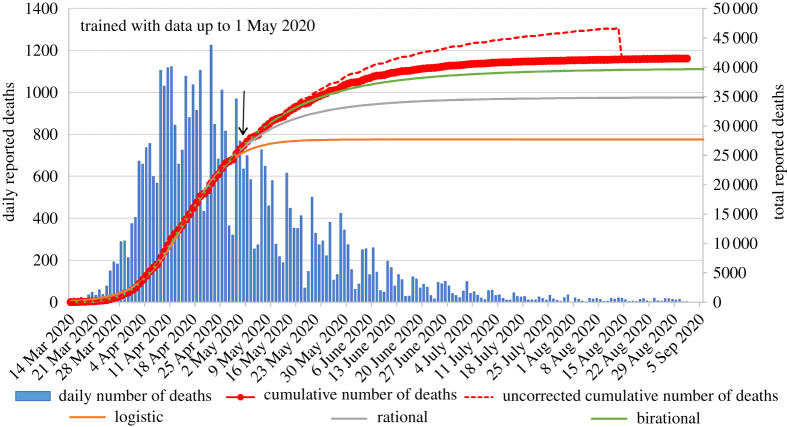
Comparison of the predictions of our formulae for the number of reported deaths for the UK using data only until 1 May 2020, versus the actual data until 1 September 2020 (predictions for 122 days). The data points with circle markers depict the cumulative number of deaths. The arrow indicates the last data point used for calibrating the formulae. Negative daily reported deaths are not displayed for clarity. The dashed curve depicts the earlier data before the correction made on 14 August 2020. The inflection point for the UK occurred on 15 April 2020. (Online version in colour.)

Figures 2–5 present the actual versus predicted cumulative number of deaths due to Covid-19 as a function of days after 25 deaths were reported, for Spain, Germany, Italy and the UK, respectively. The three formulae were trained with data up to 1 May 2020, which corresponds to T + 27, T + 25, T + 16 and T + 16, respectively. Regarding the birational formula, we used X = T. The predictions of the three formulae were compared for the following 122 days, namely until 1 September 2020. It is evident from these curves that, whereas each of the explicit logistic, rational and birational formulae *can* fit the data quite well, the predictive capacities of these formulae are *quite different*. The predictions of our two novel formulae for the 122 days following 1 May are remarkably accurate for all four counties analysed here. Regarding Spain, it is interesting to note that early on it appeared that our predictions were slightly off, but after the real data were corrected at the official site of the European Centre for Disease Prevention and Control, the curves depicting our predictions are essentially indistinguishable from the curve depicting the real data. Similarly, for the UK, our predictions differed substantially from the dashed curve (online version in red) in figure 5, which depicts the *earlier* data, but, again, after these data were officially corrected it became clear that our predictions are quite accurate.

For the epidemic in Spain (figure 2), the logistic formula predicts a plateau on 7 May 2020 (day 59 after the day that 25 deaths were reported) with 23 795 deaths; the rational and birational formulae predict a plateau on 25 May 2020 (day 77) with 26 581 and 27 090 deaths, respectively. From the actual reported data until 1 September 2020, we were able to determine that the actual plateau occurred on approximately 27 May 2020 (day 79) with approximately 27 940 deaths (owing to the repeated corrections imposed by the official agency reporting the number of Covid-19 deaths, it is not possible to identify the above numbers exactly). That is, only 2 days and 850 deaths over the estimate of the birational formula.

For the epidemic in Germany (figure 3), the logistic formula predicts a plateau on 17 May 2020 (59 days after the day that 25 deaths were reported) with 6830 deaths; the rational formula predicts a plateau on 16 June 2020 (day 89) with 8702 deaths. From the actual reported data until 1 September 2020, we were able to determine that the actual plateau for Germany occurred on 20 June 2020 (day 93) with 8882 total deaths. That is, only 4 days and 180 deaths over the estimate of the rational formula.

For the epidemic in Italy (figure 4), the logistic formula predicts a plateau on 12 May 2020 (73 days after the day that 25 deaths were reported) with 27 390 deaths; the rational formula predicts a plateau on 9 June 2020 (day 101) with 31 976 deaths; and the birational formula predicts a plateau on 19 June 2020 (day 111) with 34 399 deaths. From the actual reported data until 1 September 2020, we were able to determine that the actual plateau for Italy for the number of deaths due to Covid-19 occurred on 22 June 2020 (day 114) with 34 634 total deaths. That is, only 3 days and 235 deaths over the estimate of the birational formula.

For the epidemic in the UK (figure 5), the logistic formula predicts a plateau on 15 May 2020 (62 days after the day that 25 deaths were reported) with 27 433 deaths; the rational formula predicts a plateau on 10 June 2020 (day 88) with 33 886 deaths; and the birational formula predicts a plateau on 25 June 2020 (day 103) with 38 487 deaths. From the actual reported data until 1 September 2020 (corrected on 18 August), we were able to determine that the actual plateau for the UK for the number of deaths due to Covid-19 occurred on 28 June 2020. That is, only 3 days and 1802 deaths over the estimate of the birational formula.

## 3. Discussion

Several useful models elucidating aspects of the SARS-CoV-2 pandemic have already appeared in the literature, including [8–17]. Also, Roosa *et al.* [18,19] investigated phenomenological models to perform real-time and short-term predictions of the reported cases for the Covid-19 pandemic in China. Most of these models depend only on the time variable *t*, but in [20], where a useful analogy is presented between the spreading of an infection and the process of chemical reaction, the dependence of a space variable of the spread of infection is also taken into consideration. In [21], a novel approach is presented for reducing the cost of testing without compromising accuracy: combining subsamples and testing them in groups. The above references represent only a tiny fraction of more than *3000* publications that have appeared in the last four months in arχiv, medRχiv and bioRχiv.

Here, following [7], we modelled the cumulative number *N* of deaths caused by Covid-19 in a given country as a function of time, in terms of a Riccati equation. This equation is specified by the constant N_f_ and the function *α*(*t*). Although this Riccati equation is a nonlinear ordinary differential equation containing time-dependent coefficients, it can be solved in closed form. For appropriately chosen functions *α(t)*, the solution provides a flexible generalization of the classical logistic formula given by equation (1.1) that has been employed in a great variety of applications, including the modelling of infectious processes.

The fact that *α* is now a function of *t* has important implications. In particular, it made it possible to construct the rational and birational formulae. These two formulae, as well as the logistic formula, provide good fits for the available data. However, as discussed in detail above, the rational and birational formulae provide much more *accurate predictions.* As shown in figure 2, the success of our explicit formulae for three out of the four counties analysed here is truly remarkable. The fact that for the UK our predictions are not as good as in the other three countries suggests that our model may not be as accurate for countries which delayed the implementation of the lockdown measures. The overall unexpected success of our formulae suggests that the few constant parameters specifying the rational and birational formulae capture the essence of the epidemiological characteristics associated with the deaths caused by Covid-19. This is reminiscent of the well-known Anderson–May model, where a single parameter characterizing a constant coefficient Riccati equation captures the effect of the combination of several different biological mechanisms [22]. An additional explanation of the above success is that our approach is ‘data driven’. Indeed, the given data are used to fix the unknown parameters occurring in our mathematical formulae, and then the resulting algorithms (just as happens with the algorithms in artificial intelligence) can be used for predictions.

It is noted that our approach has the capacity for increasing continuously the accuracy of the predictions: as soon as the epidemic in a given country passes the time T, the rational model can be used; furthermore, when the sigmoidal part of the curve is approached, the rational formula can be supplemented with the birational formula. It should be noted that all three models examined in this work, namely the logistic, rational and birational formulae, do not perform well if they are calibrated with data before the epidemic peak (maximum rate or deaths, inflection point T).

Following this expected decline of the ‘first wave’ of infections after the lockdown, several European countries began easing their lockdown measures. In particular, in Spain, the initial easing of the Covid-19 measures occurred on 13 April, when workers in some non-essential sectors, such as construction and industry, were allowed to return to work; on 28 April, the government announced further plans for easing lockdown restrictions [23]. In Germany, initial easing of the Covid-19 measures occurred on 20 April, when smaller shops (shops with a retail space of up to 800 m^2^), as well as bookshops, bike stores and car dealerships, were allowed to reopen to the public [24,25]; schools as well as hair salons opened on 4 May; by 9 May, all statewide curfews had been lifted. Italy started easing the lockdown on 4 May 2020, when millions of Italians went back to work (manufacturing industries and construction sites reopened after seven weeks of restrictive measures); swimming pools, sports centres and gyms reopened on 25 May; theatres and cinemas reopened on 15 June [26]. In the UK, the government eased the lockdown measures on 10 May, by changing the slogan from ‘Stay at Home’ to ‘Stay Alert’ [27]; people could exercise more than once daily outdoors and could interact with others while maintaining social distancing. However, the situation in the UK worsened, leading the Prime Minister to postpone some lockdown easing measures scheduled to begin in England on 1 August [28].

Taking into consideration the devastating impact of Covid-19 on the economy, as well as its implications for the psychological health of the population [29], the decision for easing the lockdown measures was considered necessary. A rigorous and computationally effective approach for calculating the number of deaths following the easing of the lockdown measures is presented in [30] (the employment of the Riccati equation studied here was an important ingredient of the algorithm presented in [30]). The analysis of [30] is based on the *assumption* that the characteristics of the virus remain the same in the post-lockdown period. Under this assumption, it is shown that the numbers both of the infected reported individuals and of deaths will begin to grow in the post-lockdown period. Actually, the number of reported infected cases began growing in Spain in the second week of July, in Germany at the end of July, in Italy in the first week of August and in the UK in the second week of July. Thus, the number of reported infected cases began to grow only approximately two to three months after these countries lifted the lockdown measures. Furthermore, the increase in the number of infected individuals was *not* accompanied by an analogous increase in the number of deaths: in Italy, there is no deviation between the curve depicting the number of deaths and the curve of our predictions, whereas in Spain and Germany a small deviation began to occur in the first week of August; in the UK, where our predictions are not as accurate as in the other three countries, there is *no* increase in the deviation between data and predictions since the second week of June. The long delay in the increase in the number of reported infected individuals as well the relatively small number of deaths suggest that the virus responsible for the current epidemics in these countries may have mutated to one which is less virulent. Indeed, it is shown in [31] that the virus mutated to a variant which has the G614 form of the spike protein instead of the D614 form that was originally identified from the first human cases in Wuhan, China. However, the question of whether this form is less virulent remains open, despite a report in *The New York Times* reproduced in many newspapers (see for example [32]), where it is erroneously stated that in [31] it is actually proven that the mutated virus is less virulent. An alternative explanation for the observation that the increase in infected individuals was not immediately followed by an increase in deaths is that the early infections occurred in ‘younger’ persons; taking into consideration that the SARS-CoV-2 infection in ‘younger’ individuals rarely leads to death, and that an unknown time period is required for ‘younger’ individuals to infect ‘older’ once it follows that there should be a time delay before an increase in the number of reported infected individuals and an increase in the number of deaths. In this connection, it is noted that by extending the algorithm developed in [30] into two sub-populations consisting of ‘younger’ (below 40) and ‘older’ (above 40), it was shown in [33] regarding the epidemic in Greece that, if the number of contacts between asymptomatic ‘younger’ persons increases, the number of reported infected cases increases but not the number of deaths; on the other hand, the increase in contacts involving ‘older’ persons leads to a dramatic increase in the number of deaths. Interestingly, the increase in the number of deaths in Spain and Germany began to occur after approximately four weeks from the time that the number of reported infected cases began to grow. Independently of whether the virus has mutated to a less virulent form or not, a most beneficial factor in the fight against the pandemic is the adherence to protective measures of the vast majority of older and vulnerable individuals. The next couple of weeks following submission of our manuscript are of crucial importance for the epidemics in Spain and Germany, and hence, by analogy, for the epidemics in other European countries: if the deviations observed in figures 2 and 3 become pronounced, then there may be a second substantial wave of deaths, which will be consistent with the predictions of [30]; on the other hand, if the deviation remains small, it will imply that the epidemics will remain under control. It is natural to hypothesize that the different tempo in the four countries examined here simply reflects the fact that the lifting of lockdown measures was implemented first in Spain and then was followed by Germany, Italy and the UK.

## 4. Material and methods

We obtained the time-series data for Covid-19 for Spain, Germany, Italy and the UK from the official site of the European Centre for Disease Prevention and Control.^2^ We arranged the data in the form of deaths, *N*, over time measured in days, after the day that the number of deaths reached 25.

Throughout this paper, the unknown parameters were determined by employing the *simplex algorithm*. This is based on an iterative procedure that does not need information regarding the derivative of the function under consideration. The simplex algorithm is particularly effective for cases where the gradient of the likelihood functions is not easy to calculate. The algorithm creates a ‘random’ simplex of *n* + 1 points, where *n* is the number of model parameters that need to be estimated. The constrained variation of the simplex algorithm [34,35] available in MATLAB® was used for all models; the L_1_*-norm* was employed in the likelihood function to improve robustness [36]. Random parameter initializations were used to avoid local minima. The simplex algorithm was chosen because it performed better than certain nonlinear least-squares curve-fitting algorithms evaluated in this work, namely the Levenberg–Marquardt [37] and trust-region-reflective algorithms [38].

The stability of the fitting procedure was established by using the following simple criterion: different fitting attempts based on the use of a fixed number of data points must yield curves which have the same form beyond these fixed points. In this way, it was established that the rational formula could be employed provided that data were available until around the time T, whereas the birational formula could be used only for data available well beyond T.

The fitting accuracy of each formula was evaluated by fitting the associated formula on *all* the available data until 1 May 2020. The relevant parameters specifying the logistic, rational and birational formulae are given in table 1. Confidence intervals for each fitting parameter were calculated based on [39,40].

We *assumed* that the function *N*(*t*) satisfies the Riccati ordinary differential equation

4.1 $$\frac{\text{d}N}{\text{d}t}=\alpha (t)\left(N-\frac{N^{2}}{N_{f}}\right).$$

The general solution of this equation is given by [7]

4.2 $$N=\frac{N_{f}}{1+\beta {\text{e}}^{-\tau }},\tau =\int \alpha (t)\text{d}t,$$

where β is the constant arising from the integrating equation (4.1). In the particular case that *α*(*t*) = k, equation (4.2) becomes the logistic formula of (1.1). If *α*(*t*) is given by the rational function

$$\alpha (t)=\frac{\mathrm{kd}}{1+\mathrm{kt}},$$

then equation (4.2) becomes the rational formula

4.3 $$N=\frac{N_{f}}{1+\beta {\left(1+\text{d}t\right)}^{-k}}.$$

The birational formula is given by

4.4 $$N=\left\{ \begin{array}{l} \frac{c}{1+b{\left(1+\text{d}t\right)}^{-k}},\;t\leq X\\ \frac{c}{1+b{\left(1+\mathrm{dX}\right)}^{-k}}-\frac{c_{1}}{1+b_{1}{\left(1+d_{1}X\right)}^{-k_{1}}}+\frac{c_{1}}{1+b_{1}{\left(1+d_{1}t\right)}^{-k_{1}}},\;t>X, \end{array}\right.$$

where the fixed parameter *X* is either T or in the neighbourhood of T. For the birational model

4.5 $$\alpha (t)=\left\{ \begin{array}{l} \frac{\mathrm{kd}}{1+\text{d}t},\;t\leq X\\ \frac{k_{1}d_{1}}{1+d_{1}t}\frac{1}{1+(1-(c_{1}/N_{f})){\left(1+d_{1}t\right)}^{-k_{1}}},\;t>X. \end{array}\right.$$

Letting in equation (4.4) t→∞ we find

4.6 $$N_{f}=\frac{c}{1+b{\left(1+\mathrm{dX}\right)}^{-k}}-\frac{c_{1}}{1+b_{1}{\left(1+d_{1}X\right)}^{-k_{1}}}+c_{1}.$$

If b, c, d, k are close to b_1_, c_1,_ d_1_, k_1_, then N_f_ is close to c_1_, and hence the value of *α*(*t*) after *t* = X is close to the value of *α*(*t*) before *t* = X.

The constant T can be computed by solving the equation obtained by equating to zero the second derivative of *N*. This implies that for the logistic and the rational models T is given, respectively, by

4.7 $$T=\frac{\ln(\beta )}{k},\;{\left(1+\mathrm{dT}\right)}^{k}=b\left(\frac{k-1}{k+1}\right).$$

Similarly, for the birational model where the parameters b, d and k in the right-hand equation of equations (4.7) are replaced by b_1_, d_1_ and k_1_, respectively.

Our work uses only the logistic, rational and birational formulae, namely equations (1.1), (4.3) and (4.4) with X = T, respectively.

## Footnotes

1 https://www.fda.gov/news-events/press-announcements/coronavirus-covid-19-update-fda-issues-emergency-use-authorization-potential-covid-19-treatment.

2 https://www.ecdc.europa.eu/en/geographical-distribution-2019-ncov-cases.

## Data accessibility

The time-series data for Covid-19 were obtained from the official site of the European Centre for Disease Prevention and Control.
